# Homologous haplotypes, expression, genetic effects and geographic distribution of the wheat yield gene *TaGW2*

**DOI:** 10.1186/1471-2229-14-107

**Published:** 2014-04-25

**Authors:** Lin Qin, Chenyang Hao, Jian Hou, Yuquan Wang, Tian Li, Lanfen Wang, Zhengqiang Ma, Xueyong Zhang

**Affiliations:** 1 Key Laboratory of Crop Gene Resources and Germplasm Enhancment, Ministry of Agriculture/The National Key Facility for Crop Gene Resources and Genetic Improvement/Institute of Crop Science, Chinese Academy of Agricultural Sciences, Beijing 100081, China; 2 Crop Genomics and Bioinformatics Center and National Key Lab of Crop Genetics and Germplasm Enhancement, Nanjing Agricultural University, Nanjing 210095, Jiangsu, China

**Keywords:** *Triticum aestivum*, *TaGW2*, Grain weight, Gene expression, Haplotype interaction

## Abstract

**Background:**

*TaGW2-6A*, cloned in earlier research, strongly influences wheat grain width and TKW. Here, we mainly analyzed haplotypes of *TaGW2-6B* and their effects on TKW and interaction with haplotypes at *TaGW2-6A.*

**Results:**

About 2.9 kb of the promoter sequences of *TaGW2-6B* and *TaGW2-6D* were cloned in 34 bread wheat cultivars. Eleven SNPs were detected in the promoter region of *TaGW2-6B,* forming 4 haplotypes, but no divergence was detected in the *TaGW2-6D* promoter or coding region. Three molecular markers including CAPS, dCAPS and ACAS, were developed to distinguish the *TaGW2-6B* haplotypes. Haplotype association analysis indicated that *TaGW2-6B* has a stronger influence than *TaGW2-6A* on TKW, and *Hap-6B-1* was a favored haplotype increasing grain width and weight that had undergone strong positive selection in global wheat breeding. However, clear geographic distribution differences for *TaGW2-6A* haplotypes were found; *Hap-6A-A* was favored in Chinese, Australian and Russian cultivars, whereas *Hap-6A-G* was preferred in European, American and CIMMYT cultivars. This difference might be caused by a flowering and maturity time difference between the two haplotypes. *Hap-6A-A* is the earlier type. Haplotype interaction analysis between *TaGW2-6A* and *TaGW2-6B* showed additive effects between the favored haplotypes. *Hap-6A-A/Hap-6B-1* was the best combination to increase TKW. Relative expression analysis of the three *TaGW2* homoeologous genes in 22 cultivars revealed that *TaGW2-6A* underwent the highest expression. *TaGW2-6D* was the least expressed during grain development and *TaGW2-6B* was intermediate. Diversity of the three genes was negatively correlated with their effect on TKW.

**Conclusions:**

Genetic effects, expression patterns and historic changes of haplotypes at three homoeologous genes of *TaGW2* influencing yield were dissected in wheat cultivars. Strong and constant selection to favored haplotypes has been found in global wheat breeding during the past century. This research also provides a valuable case for understanding interaction of genes that control complex traits in polyploid species.

## Background

Common wheat is a hexaploid species (AABBDD) with a large genome size (17.9 × 10^9^ bp) and abundant repeat sequences (>80%) [1]. Comparative genomics proved the existence of genomic colinearity among cereal crops [2]. As a model plant of cereals, the rice genomic sequence completed in 2002 [3,4], and several yield-related genes [5,6], such as *GS3*, *GW2*, *GW5*, *GW8*, *TGW6*, *Ghd7* and *GIF1*, have been isolated [7-13], providing opportunities for homology-based cloning of yield-related genes in other cereals. The availability of a draft wheat genome sequence [14-17] will promote genome-based research of this extremely important crop. Cloning yield-related genes, exploring the favored alleles and developing functional markers will be important for yield improvement in that crop. This will be the next major focus of wheat genetics and genomics.

Among yield-related genes, current studies on gene function and allele discovery of *GW2* are the most in-depth and extensive in cereal crops. Firstly, Song *et al.*[8] isolated a major yield QTL from rice, which was mapped on short arm of chromosome 2 and designated as *OsGW2*. It encoded a RING-type protein with E3 ubiquitin ligase activity that negatively regulated grain width, and loss-of-function mutations enhanced grain weight and yield. In maize, Li *et al.* (2010) [18] found two homologs of *OsGW2*, viz. *ZmGW2-CHR4* and *ZmGW2-CHR5*, and a SNP in the promoter region of *ZmGW2-CHR4* was significantly associated with kernel width (KW) and hundred kernel weight (HKW) in maize. We cloned *TaGW2* from chromosome 6A of wheat, and found SNPs in its promoter region, that were significantly associated with KW and TKW. A CAPS marker was developed based on the -593 A/G polymorphism and association analysis indicated that *Hap-6A-A* increased TKW by more than 3.1 g [19]. Recently, a *TaGW2-6A*-CAPS marker was used to detect variation in a BC_2_F_4_ RIL population, as well as a natural population, further demonstrating that *TaGW2-6A* was significantly associated with grain weight [20]. Yang *et al.*[21] identified a single-base insertion in the eighth exon of *TaGW2-6A* causing premature termination in landrace Lankaodali, which ultimately led to increased grain width and grain weight. However, Bednarek *et al.*[22] showed that the patterns of *TaGW2* regulation of grain development might be more complex after studies on RNA interference (RNAi) of expression of *TaGW2* in wheat. In consideration of the characteristics of the wheat genome, further dissection of the regulation and expression patterns of the three *TaGW2* homoeologous genes on grain weight could have important biological and breeding implications.

In this study, further research focused on sequencing and diversity studies of the promoter regions of *TaGW2-6B* and *TaGW2-6D*, functional marker development, and an expression pattern comparison of the three homoeologous *TaGW2* loci. Hence, the major objectives were to (1) reveal sequence diversity and distribution characteristics of the three *GW2* homoeologous genes by sequence alignment of their ~2.9 kb promoter regions; (2) develop functional markers for *TaGW2-6B* and *TaGW2-6D* to distinguish various haplotypes, and discover favored haplotypes for yield improvement through association analysis; (3) evaluate the distributions of different haplotypes in global wheat major production regions, including North America, Europe, Australia, Russia, Mexico and China, and understand the selection intensity and geographical distribution of *TaGW2s* in different wheat ecological regions; (4) assess the relationships between the expression levels of the three *TaGW2* homoeologues and grain size by real-time PCR analysis, and preliminarily evaluate the genetic effects of *TaGW2s* based on phenotypic variation (*R*^*2*^) for grain traits; and (5) examine interactions among the three *TaGW2* loci on chromosomes 6A, 6B and 6D through haplotype combination analysis. It was expected that the study would identify important genes and functional markers for wheat yield improvement.

## Results

### Major variations in *TaGW2s* occur in the promoter regions

**Figure 1:**
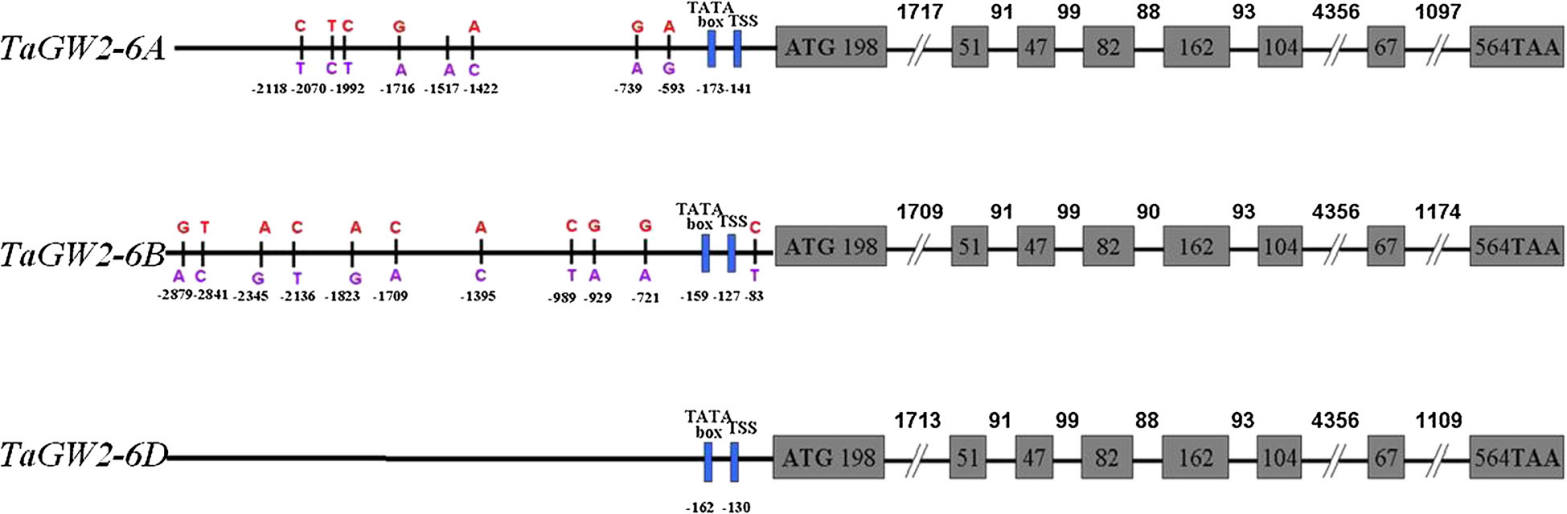
**Gene structures of *TaGW2-6A, -6B *and *-6D.*** Variations mainly occurred in the promoter regions.

**Figure 2:**
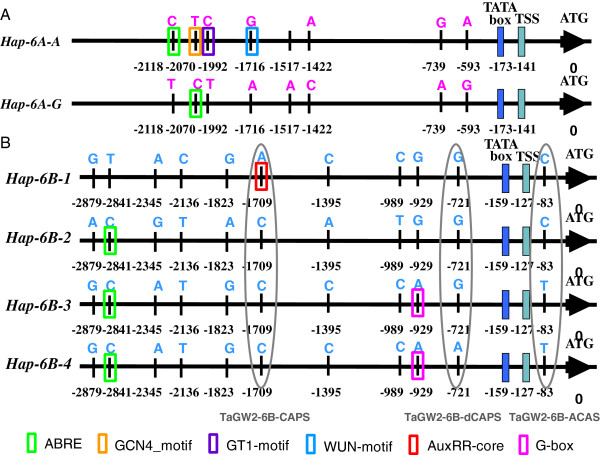
**Haplotypes and predicted *cis*-acting regulatory elements in the promoter regions of *TaGW2-6A *and *TaGW2-6B. *A**, two haplotypes were formed by 8 SNPs in the *TaGW2-6A* promoter region. **B**, four haplotypes were formed by 11 SNPs in the *TaGW2-6B* promoter region. The ellipses mean the polymorphic sites where markers were developed. The rectangles mean cis-acting regulatory elements. ABRE, abscisic acid-responsive element; GCN4_motif, endosperm tissue-specific expression; GT1-motif, light responsive element; WUN-motif, wound responsive element; AuxRR-core, auxin responsive element; G-box, light responsive element.

In the coding sequence of *TaGW2* homoeologous genes, 34 wheat accessions (Additional file 1: Table S3) were used to study the nucleotide polymorphism and no divergence was found. Genome walking was used to clone the sequences of the promoter regions of *TaGW2-6B* and *TaGW2-6D*, and ~2.9 kb upstream sequences from the ATG start codons were obtained. The core elements of the promoters were predicted with the TSSP program (http://www.softberry.com), and the TATA box and STS (Start Transcription Site) were identified at -159 bp and -127 bp upstream from the ATG codon of *TaGW2-6B*. For *TaGW2-6D*, the corresponding locations were located at -162 bp and -130 bp, respectively. Generally, more variations in *TaGW2s* occurred in the promoter regions, but the diversity of *TaGW2-6B* was higher than that of *TaGW2-6A*, in which eight SNPs forming two haplotypes were found earlier [19]. No divergence was detected in the *TaGW2-6D* promoter region (Figure 1). Four haplotypes were formed by 11 SNPs within the 2.9 kb upstream sequence of *TaGW2-6B;* these were designated *Hap-6B-1*, *Hap-6B-2*, *Hap-6B-3* and *Hap-6B-4* (Figure 2).

### Haplotypes in promoter region of *TaGW2-6B* have strong effects on TKW

#### *TaGW2-6B marker development*

**Figure 3:**
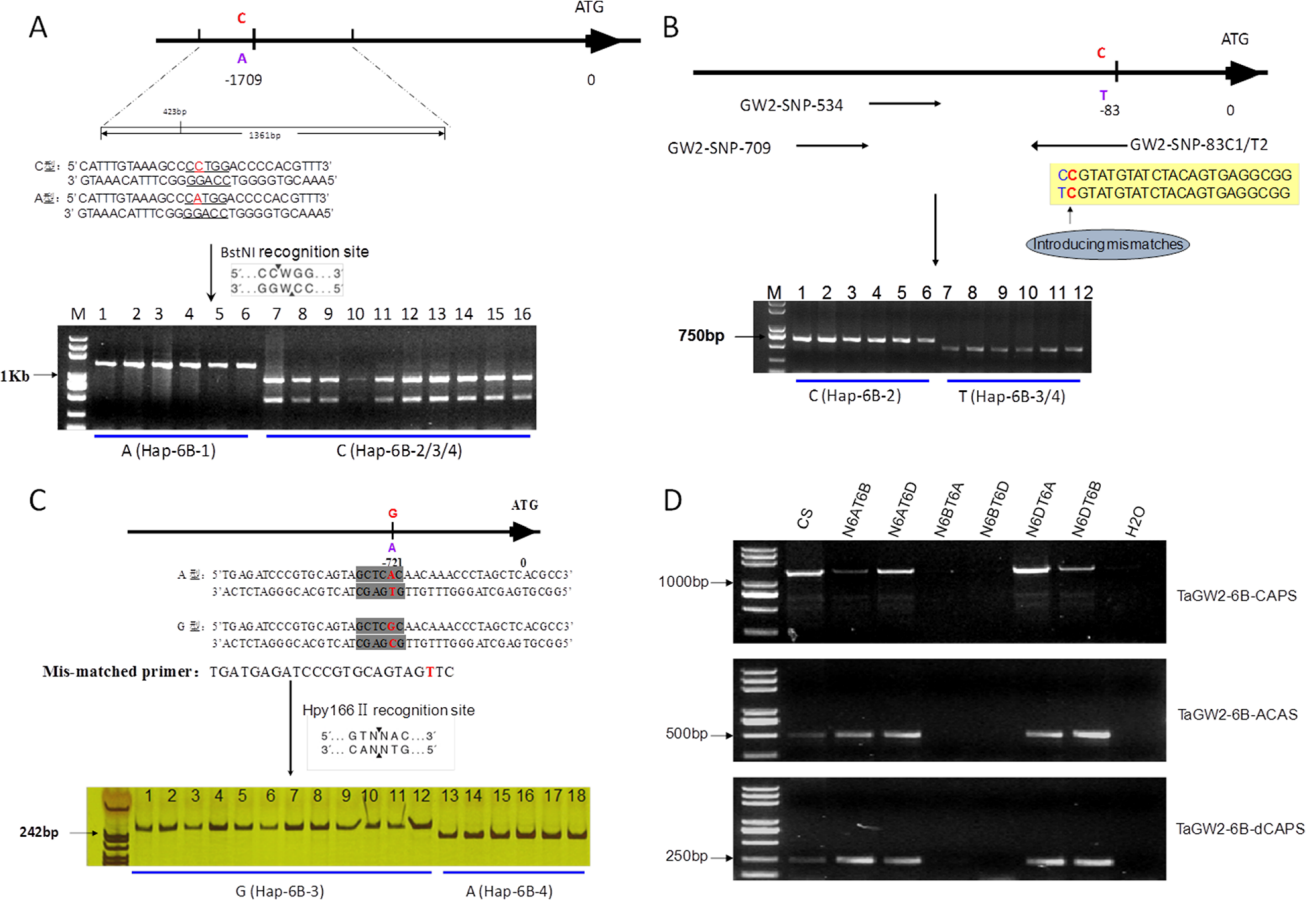
**Marker development and genetic mapping of *TaGW2-6B. *A**, CAPS marker was developed using nucleotide polymorphism at -1709 bp; **B**, ACAS-PCR marker was designed for SNP-83 T/C; **C**, dCAPS marker was based on one SNP difference at -721 bp; **D**, All of the markers based on polymorphisms in the upstream region of *TaGW2-6B* were mapped on chromosome 6B in common wheat. All wheat accessions used in this study for developing markers were listed in Additional file 1: Table S3.

In the 11 SNPs detected in the *TaGW2-6B* promoter region (Figure 2), the nucleotide polymorphism at -1709 bp created a restriction enzyme recognition site for *BstNI* (CCWGG) (Figure 3A). This was employed to develop a cleaved amplified polymorphism sequence (CAPS) marker to distinguish *Hap-6B-1* from the other three haplotypes. No restriction enzyme recognition site was found in *Hap-6B-1* (-1709A), whereas it existed in the other three haplotypes (-1709C). In addition, ACAS-PCR primer sets designed for SNP-83 T/C worked well and were co-dominant (Figure 3B). The forward primer for ACAS-PCR was genome-specific, and the reverse was allele-specific with artificial mismatches in the 3′-end. *Hap-6B-1* and *Hap-6B-2* amplified a fragment of 626 bp, whereas *Hap-6B-3* and *Hap-6B-4* amplified a 464 bp fragment. Thus, the ACAS-PCR primer sets reliably discriminated *Hap-6B-2* and the other two haplotypes. Finally, only one SNP difference was found at -721 bp for discriminating *Hap-6B-3* and *Hap-6B-4*. The dCAPS marker was designed with a specific mismatch in the primer to introduce a restriction enzyme Hpy166II recognition site (Figure 3C) using an available program dCAPS Finder 2.0 (http://helix.wustl.edu/dcaps/dcaps.html). This marker effectively discriminated *Hap-6B-3* (263 bp) and *Hap-6B-4* (240 bp). Thus, three markers, *TaGW2*-6B-CAPS, *TaGW2*-6B-dCAPS and *TaGW2*-6B-ACAS, were developed to distinguish these haplotypes.

Tests on a set of Chinese Spring (CS) nullisomic-tetrasomic lines confirmed that the three markers were chromosome 6B-specific (Figure 3D). The *TaGW2-6B* gene was mapped between the markers *Xmag359* and *Xwmc341* on chromosome 6B in the recombinant inbred line (RIL) population derived from Nanda 2419 and Wangshuibai (Additional file 2: Figure S1). Based on the wheat consensus SSR genetic map [23], *TaGW2-6B* was very close to the 6B centromere.

### Strong differences in TKW and heading date exist between *TaGW2-6B* haplotypes

All three molecular markers, distinguishing the four *TaGW2-6B* promoter haplotypes were used for genotyping the 265 entries in the Chinese wheat mini-core collection. Previous studies had demonstrated that these accessions were clustered into two sub-populations comprising 151 landraces and 114 modern cultivars [24,25] by Structure v2.1 software [26]. Therefore, association analysis between haplotypes of *TaGW2-6B* and grain traits took population structure into account.

There were significant differences in TKW between *Hap-6B-1* and *Hap-6B-4* in the landraces (*P <0.01* in 2002, *P <0.05* in 2006), and phenotypic differences between them were 6.38 g and 4.68 g in 2002 and 2006, respectively (Table 1). This might be caused by differences in KL between *Hap-6B-1* and *Hap-6B-4* (0.43 mm in 2002, 0.49 mm in 2006). Among modern cultivars, significant differences were again detected in TKW between *Hap-6B-1* and *Hap-6B-4* (*P <0.01* in 2002 and 2006), and the mean TKW differences of *Hap-6B-1* and *Hap-6B-4* were 16.68 g and 15.25 g. These differences were due to large differences in KW and KT (Table 1). KW differences between the two groups were 0.45 mm and 0.39 mm, the KT differences were 0.45 mm and 0.33 mm, respectively. The significant negative effect of *Hap-6B-4* may be the major reason for its elimination in breeding. Compared with the other three haplotypes, *Hap-6B-1* was the favored one that increased grain weight. It was noteworthy that *Hap-6B-2* was quite close to *Hap-6B-1* in effect on grain weight in modern Chinese cultivars.

**Table 1:** ***TaGW2-6B *** **haplotype associations with grain traits in two environments**

**Trait/genotype**	**02LY**				**06LY**			
	***Hap-6B-1***	***Hap-6B-2***	***Hap-6B-3***	***Hap-6B-4***	***Hap-6B-1***	***Hap-6B-2***	***Hap-6B-3***	***Hap-6B-4***
**Overall**								
KL (mm)	6.77 ± 0.06a (A)	6.58 ± 0.06ab (AB)	6.40 ± 0.05bc(B)	6.27 ± 0.09c(B)	6.73 ± 0.05a(A)	6.56 ± 0.06a(AB)	6.35 ± 0.06b(BC)	6.15 ± 0.08b(C)
KW (mm)	3.23 ± 0.02a(A)	3.15 ± 0.03a(A)	3.04 ± 0.02b(B)	2.91 ± 0.04c(B)	3.29 ± 0.02a(A)	3.19 ± 0.02b(B)	3.11 ± 0.02c(BC)	3.05 ± 0.03c(C)
KT(mm)	2.90 ± 0.02a(A)	2.84 ± 0.02ab(AB)	2.79 ± 0.02b(BC)	2.67 ± 0.04c(C)	2.93 ± 0.02a(A)	2.86 ± 0.02b(AB)	2.80 ± 0.02bc(B)	2.74 ± 0.03c(B)
KL/KW ratio	2.10 ± 0.02a	2.09 ± 0.02a	2.11 ± 0.02a	2.16 ± 0.04a	2.05 ± 0.02a	2.06 ± 0.02a	2.05 ± 0.02a	2.02 ± 0.03a
TKW (g)	40.39 ± 0.71a(A)	36.72 ± 0.88b(B)	33.89 ± 0.72c(BC)	29.35 ± 0.89d(C)	40.99 ± 0.72a(A)	36.94 ± 0.83b(B)	33.88 ± 0.60c(BC)	31.20 ± 0.82c(C)
**Landraces**								
KL (mm)	6.70 ± 0.16a	6.38 ± 0.08ab	6.36 ± 0.06b	6.27 ± 0.10b	6.64 ± 0.13a(A)	6.38 ± 0.07ab(AB)	6.33 ± 0.06ab(AB)	6.15 ± 0.09b(B)
KW (mm)	3.01 ± 0.05a	3.01 ± 0.03a	3.01 ± 0.02a	2.92 ± 0.04a	3.13 ± 0.05a	3.11 ± 0.02a	3.10 ± 0.02a	3.06 ± 0.03a
KT (mm)	2.79 ± 0.05a	2.77 ± 0.03a	2.77 ± 0.02a	2.69 ± 0.04a	2.83 ± 0.04a	2.79 ± 0.03a	2.78 ± 0.02a	2.75 ± 0.03a
KL/KW ratio	2.23 ± 0.05a	2.12 ± 0.03a	2.12 ± 0.02a	2.16 ± 0.05a	2.13 ± 0.04a	2.06 ± 0.03a	2.05 ± 0.02a	2.01 ± 0.03a
TKW (g)	36.08 ± 2.03a(A)	32.44 ± 1.02ab(AB)	33.06 ± 0.70ab(AB)	29.70 ± 0.90b(B)	36.21 ± 1.76a	32.50 ± 0.79b	33.29 ± 0.60ab	31.53 ± 0.81b
**Modern cultivars**								
KL (mm)	6.79 ± 0.06a	6.80 ± 0.08a	6.63 ± 0.09a	6.22 ± 0.02a	6.76 ± 0.05a	6.76 ± 0.10a	6.48 ± 0.15a	6.13 ± 0.03a
KW (mm)	3.30 ± 0.02a(A)	3.30 ± 0.03a(A)	3.23 ± 0.06a(AB)	2.85 ± 0.10b(B)	3.34 ± 0.02a	3.29 ± 0.03ab	3.21 ± 0.04ab	2.95 ± 0.15b
KT (mm)	2.94 ± 0.02a(A)	2.90 ± 0.03a(A)	2.91 ± 0.05a(A)	2.49 ± 0.11b(B)	2.96 ± 0.02a	2.94 ± 0.02a	2.91 ± 0.05ab	2.63 ± 0.13b
KL/KW ratio	2.06 ± 0.02a	2.06 ± 0.02a	2.06 ± 0.04a	2.19 ± 0.08a	2.03 ± 0.02a	2.06 ± 0.03a	2.02 ± 0.04a	2.09 ± 0.12a
TKW (g)	41.77 ± 0.59a(A)	41.32 ± 1.08a(A)	39.71 ± 2.16a(A)	25.09 ± 3.62b(B)	42.52 ± 0.67a(A)	41.70 ± 1.09a(A)	37.98 ± 1.81ab(AB)	27.27 ± 4.39b(B)

02LY, Luoyang (2002); 06LY, Luoyang (2006).

Different capital and small letters within groups indicate significance differences between haplotypes at *P* <0.01 and *P* <0.05 for each trait, respectively.

*Hap-6B-1, landraces, *N* = 20; Modern cultivars, *N* = 62.

*Hap-6B-2, landraces, *N* = 44; Modern cultivars, *N* = 41.

*Hap-6B-3, landraces, *N* = 63; Modern cultivars, *N* = 9.

*Hap-6B-4, landraces, *N* = 24; Modern cultivars, *N* = 2.

In addition to kernel weight, haplotype association analyses of heading and maturity dates were also performed (Additional file 3: Figure S2). There were no significant differences between *Hap-6B-1* and *Hap-6B-4* among the landraces for the two traits, but among modern cultivars heading and maturity date differences between *Hap-6B-1* and *Hap-6B-4* in both growing seasons were significant. The heading dates in 2002 and 2006 differed by 13 and 9 days and the corresponding differences for maturity date were 15 and 6 days, respectively. Similarly, *Hap-6B-2* was also 11 and 6 days earlier than *Hap-6B-4* in heading in the two seasons. For maturity, *Hap-6B-2* was 13 and 4 days earlier than *Hap-6B-4* in the two seasons respectively. Therefore, it seemed that *Hap-6B-1* and *Hap-6B-2* were associated not only with larger grain, but also earlier maturity.

### Geographic distribution and frequency changes among haplotypes of *TaGW2-6A*, and *TaGW2-6B* in global wheat breeding

#### *Geographic distribution of TaGW2-6B haplotypes in Chinese wheats*

**Figure 4:**
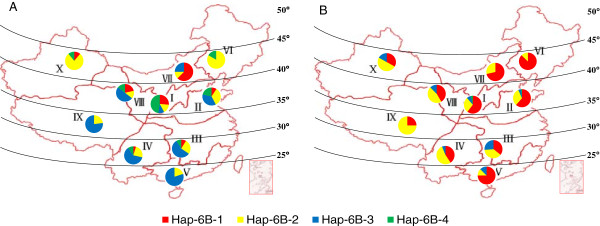
***TaGW2-6B *haplotype distribution in ten Chinese wheat ecological regions. A**, *TaGW2-6B* haplotype distribution in 151 Chinese landraces; **B**, *TaGW2-6B* haplotype distribution in 320 modern cultivars. I, Northern winter wheat region; II, Yellow and Huai River valley winter wheat region; III, Low and middle Yangtze River valley winter wheat region; IV, Southwestern winter wheat region; V, Southern winter wheat region; VI, Northeastern spring wheat region; VII, Northern spring wheat region; VIII, Northwestern spring wheat region; IX, Qinghai-Tibet spring-winter wheat region; X, Xinjiang winter-spring wheat region.

Wheat production in China is divided into ten ecological zones based on cultivar ecotypes, growing season, and cultivar response to temperature and photoperiod [25,27]. The distribution of *TaGW2-6B* haplotypes was evaluated in both landraces and modern cultivars from each zone (Figure 4). Among landraces, selection pressure on haplotypes in the different zones was not as strong as expected, and the frequency of the favored haplotype *Hap-6B-1* was generally low. In the winter wheat zones III, IV, V and IX, the frequency of *Hap-6B-3* was highest, whereas in spring wheat zones VI and X, *Hap-6B-2* was more frequent, and *Hap-6B-1* was relatively frequent only in zone VII. However, in modern cultivars, *Hap-6B-1* frequencies were higher across all zones (up to 90%), indicating it had undergone strong positive selection during wheat improvement. In detail, *Hap-6B-1* was the most frequent haplotype in zones II, V, VI and VII, whereas *Hap-6B-2* was most frequent in IV, VIII, IX and X. Association analysis showed that grain size and component parameters of *Hap-6B-2* were significantly higher than those of *Hap-6B-4*, although they were lower than those of *Hap-6B-1* (Table 1). Compared with landraces, *Hap-6B-1* and *Hap-6B-2* frequencies were higher across the ecological zones, presumably due to selective breeding, hence becoming the most frequent haplotypes. In contrast, the frequencies of *Hap-6B-3* and *Hap-6B-4* significantly decreased and even disappeared in zones IX, VI and VII (Figure 4).

**Figure 5:**
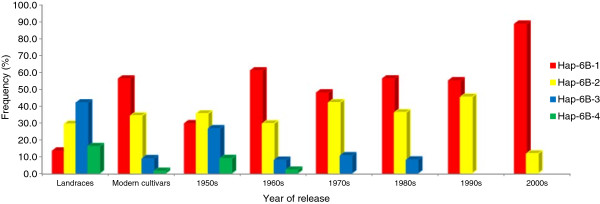
**Haplotype frequencies of *TaGW2-6B *** **in 151 landraces and 320 modern cultivars released in different periods in China.**

Further evidence showing that *TaGW2*-6B underwent strong selection in Chinese wheat breeding is provided in Figure 5. The frequency of *Hap-6B-1* showed an increasing trend, especially in the 2000s (frequencies higher than 90%). Thus this haplotype tended towards fixation during modern breeding. In contrast, *Hap-6B-4* and *Hap-6B-3* disappeared from cultivars released after the 1980s.

### Global distributions of haplotypes for *TaGW2-6A* and *TaGW2-6B*

**Figure 6:**
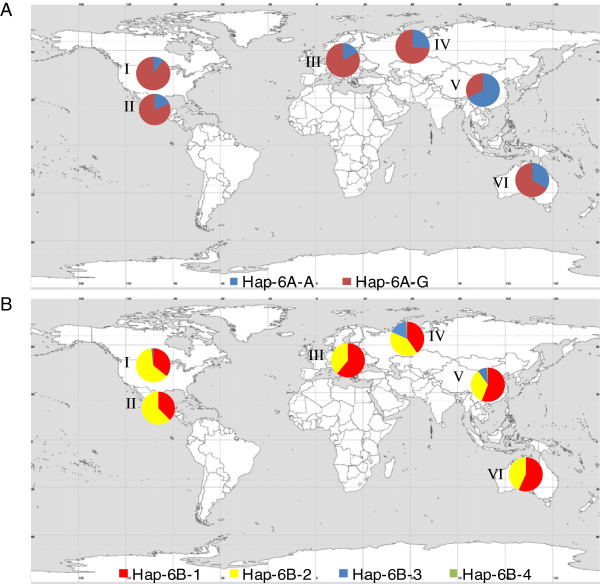
**Geographic distribution of haplotypes at *TaGW2-6A *haplotypes and *TaGW2-6B *in global wheat cultivars. A**, Geographic distribution of *TaGW2-6A* haplotypes in 320 Chinese, 374 European, 471 American, 51 Australian, 53 CIMMYT and 83 Russian accessions; **B**, Geographic distribution of *TaGW2-6B* haplotypes. North America; II, CIMMYT; III, Europe; IV, Former USSR; V, China; VI, Australia.

Previous study showed that *Hap-6A-A* was favored in China, whereas *Hap-6A-G* was favored in Europe [19]. In order to evaluate the distribution of all *TaGW2* haplotypes in global wheat cultivars, the frequencies of haplotypes at the *TaGW2-6A* and *TaGW2-6B* loci were determined in cultivar collections from North America, Australia, China, CIMMYT, Europe and Russia (Figure 6).

Obvious geographic differences in haplotype frequencies for *TaGW2-6A* were found among the different groups. *Hap-6A-A* was more frequent in Australian, Chinese and Russian cultivars, whereas *Hap-6A-G* predominated in U.S., CIMMYT and European collections (Additional file 4: Figure S3). At *TaGW2-6B*, the superior haplotype *Hap-6B-1* was more frequent in all regions, and *Hap-6B-4* was virtually absent in all groups. Selection pressure on *Hap-6A-A* in North America and Europe was apparently very low, in contrast to China, and *Hap-6A-G* tended to dominate (Additional file 4: Figure S3A-B). The favored haplotype *Hap-6B-1* at *TaGW2-6B* showed a slow growth trend, while *Hap-6B-4* gradually decreased or disappeared in all continents (Additional file 4: Figure S3C-D). Therefore, an obvious consistency of globally favored haplotypes was detected at *TaGW2-6B*, but not at *TaGW2-6A*.

### *TaGW2* genes negatively regulate wheat grain weight

**Figure 7:**
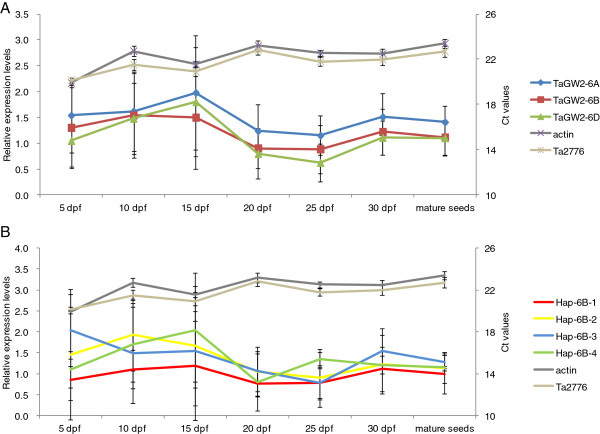
**Mean relative expression levels of *TaGW2 *in 22 wheat accessions at different stages of grain development. A**, Mean relative expression levels of *TaGW2-6A*, *TaGW2-6B* and *TaGW2-6D*; **B**, Mean relative expression levels of haplotypes of *TaGW2-6B*. First Y-axis means relative expression levels of *TaGW2*. Secondary Y-axis means average Ct values of actin and *Ta2776* genes in cultivars at different stages of grain development. As endogenous control, the actin and Ta2776 genes were not varying too much at different stages. Normalized values of *TaGW2* relative expression to actin were given as Mean ± SD.

The average expression level of *TaGW2-6A* reached a peak at 15 dpf and was significantly higher than that of either *TaGW2-6B* or *TaGW2-6D* in all six sampling stages of seed development (Figure 7A). The average relative expression of *TaGW2-6B* peaked at 10 dpf, and that of *TaGW2-6D* was 15 dpf. The average relative expression level of *TaGW2-6B* was higher than that of *TaGW2-6D* in all six stages except 15 dpf.

Differences in average relative expression of *TaGW2 genes* were detected between the 10 higher-TKW cultivars and 12 lower-TKW genotypes. Relative expression of all *TaGW2*s in the lower-TKW group peaked at 15 dpf. In the other group, *TaGW2-6A* and *TaGW2-6D* also peaked at 15 dpf, but *TaGW2-6B* peaked at 10 dpf (Additional file 5: Figure S4). Interestingly, the average relative expression level of the three *TaGW2* homoeologous genes in cultivars with lower TKW was higher than that of higher-TKW genotypes in developing seeds, whereas only small differences occurred in mature seeds (Additional file 5: Figure S4). This further confirmed that all three *TaGW2* homoeologous genes negatively regulated grain weight.

Association analysis showed that haplotypes *Hap-6A-A* and *Hap-6B-1* and *Hap-6B-2* at *TaGW2-6B* were significantly associated with higher TKW, whereas *Hap-6A-G* and *Hap-6B-4* were associated with lower TKW [19] (Table 1). The same set of 22 cultivars was used for further analysis of the relationship between relative expression levels of various *TaGW2-6A* and *TaGW2-6B* haplotypes and kernel traits (Additional file 6: Figure S5, Figure 7B). As shown in (Additional file 6: Figure S5), the average relative expression level of *Hap-6A-G* was higher than that of *Hap-6A-A* at all periods except 25 dpf, and was also very obvious at 15 dpf (approximately 1.8 times higher). The average relative expression of *Hap-6B-1* was lower than other haplotypes (Figure 7B), especially at 15 dpf. All of these results further suggested that *TaGW2s* negatively regulate grain weight by controlling the gene expression level during seed development.

### Additive genetic effects between favored haplotypes at *TaGW2-6A* and *TaGW2-6B*

**Figure 8:**
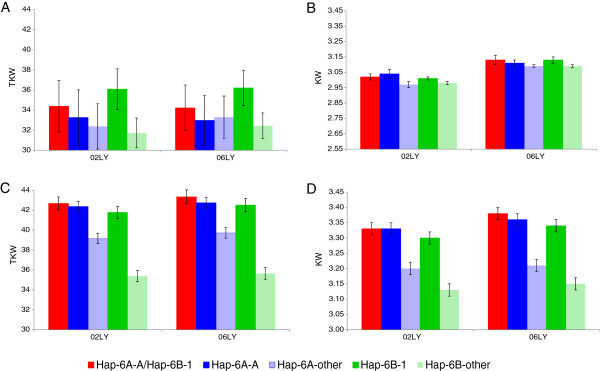
**TKWs and kernel widths for the *Hap-6A-A/Hap-6B-1 *combination and for single haplotypes. A** and **B**: landraces; **C** and **D**: modern cultivars. The bars represent the standard deviation.

To reveal combination effects between haplotypes at *TaGW2-6A* and *TaGW2-6B*, an analysis was carried out on the 265 accessions mainly coming from the Chinese wheat mini core collection (Additional file 7: Figure S6, Table 2). Eight combinations of *TaGW2-6A* and *TaGW2-6B* haplotypes were detected in landraces, but there were only seven in modern cultivars, the exception was *Hap-6A-A*/*Hap-6B-4* (*A/4*). No significant phenotypic differences were detected among these combination types in landraces (Table 2). In modern cultivars, there were significant differences between *A/1* (*Hap-6A-A*/*Hap-6B-1*) and *G/4* (*Hap-6A-G*/*Hap-6B-4*) on KT, KW and TKW, and combination *A/1* was a favored type, consistent with the earlier results [19] (Table 1, Figure 8). Combination *A/2* (*Hap-6A-A*/*Hap-6B-2*) was close to *A/1*, and much higher in TKW than *G/4*. Comparative analysis of phenotypic effects among the favored combination and superior single and other haplotypes (Figure 8) further revealed that these homoeologous genes had a strong additive effect on KW and TKW. Moreover, the favored haplotype combination *A/1* occurred at a higher frequency in the modern cultivars than in landraces, whereas small grained G/4 was the opposite (Additional file 8: Figure S7). These results indicate that combination *A/1* had undergone strong positive selection in wheat breeding due to its positive effect on grain size.

**Table 2:** **Effects of *TaGW2-6A *** **and *TaGW2-6B *** **haplotype interaction on grain traits in two environments**

**Trait/genotype**	**02LY**							
	***A/1***	***A/2***	***A/3***	***A/4***	***G/1***	***G/2***	***G/3***	***G/4***
**Overall**								
KL (mm)	6.79 ± 0.07a(A)	6.66 ± 0.08abc(AB)	6.36 ± 0.06bc(B)	6.11 ± 0.17abc(AB)	6.73 ± 0.11 ac(AB)	6.51 ± 0.09abc(AB)	6.44 ± 0.08abc(AB)	6.31 ± 0.10c(B)
KW (mm)	3.28 ± 0.03a(A)	3.25 ± 0.03a(A)	3.05 ± 0.03b(BC)	3.05 ± 0.03abc(ABC)	3.15 ± 0.04ab(AB)	3.06 ± 0.03b(BC)	3.03 ± 0.03bc(BC)	2.88 ± 0.04c(C)
KT (mm)	2.91 ± 0.03a(A)	2.89 ± 0.03ab(A)	2.78 ± 0.03b(AB)	2.86 ± 0.05abc(AB)	2.89 ± 0.03ab(A)	2.79 ± 0.03b(AB)	2.80 ± 0.03ab(AB)	2.63 ± 0.04c(B)
KL/KW ratio	2.08 ± 0.02ab	2.05 ± 0.02a	2.09 ± 0.02ab	2.00 ± 0.04ab	2.15 ± 0.03ab	2.13 ± 0.03ab	2.13 ± 0.03ab	2.20 ± 0.05b
TKW (g)	41.33 ± 0.80a(A)	39.35 ± 1.25a(ACD)	33.57 ± 0.91bc(BE)	33.34 ± 1.64abc(ABE)	38.98 ± 1.28ab(AB)	34.59 ± 1.16b(BCE)	34.35 ± 1.17b(BDE)	28.39 ± 0.93c(E)
**Landraces**								
KL (mm)	6.71 ± 0.21a	6.40 ± 0.11a	6.33 ± 0.07a	6.11 ± 0.17a	6.70 ± 0.23a	6.37 ± 0.10a	6.41 ± 0.09a	6.32 ± 0.12a
KW (mm)	3.02 ± 0.05ab(AB)	3.11 ± 0.05a(A)	3.01 ± 0.03ab(AB)	3.05 ± 0.03ab(AB)	3.01 ± 0.08ab(AB)	2.97 ± 0.03ab(AB)	3.01 ± 0.03ab(AB)	2.88 ± 0.04b(B)
KT (mm)	2.67 ± 0.07ab	2.79 ± 0.06ab	2.76 ± 0.03ab	2.86 ± 0.05ab	2.87 ± 0.06a	2.76 ± 0.04ab	2.79 ± 0.03ab	2.64 ± 0.05b
KL/KW ratio	2.23 ± 0.08a	2.06 ± 0.04a	2.11 ± 0.02a	2.00 ± 0.04a	2.23 ± 0.06a	2.15 ± 0.03a	2.13 ± 0.03a	2.20 ± 0.05a
TKW(g)	34.38 ± 2.55ab(AB)	34.33 ± 1.76ab(AB)	32.63 ± 0.84ab(AB)	33.34 ± 1.64ab(AB)	37.22 ± 2.96a(A)	31.64 ± 1.24ab(AB)	33.68 ± 1.22ab(AB)	28.74 ± 0.95b(B)
**Modern cultivars**								
KL (mm)	6.81 ± 0.08a	6.79 ± 0.10a	6.59 ± 0.16a		6.75 ± 0.11a	6.80 ± 0.15a	6.68 ± 0.05a	6.22 ± 0.02a
KW (mm)	3.33 ± 0.02a(A)	3.33 ± 0.04a(A)	3.29 ± 0.06ab(AB)		3.23 ± 0.04ab(AB)	3.24 ± 0.05ab(AB)	3.15 ± 0.09ab(AB)	2.85 ± 0.10b(B)
KT (mm)	2.96 ± 0.02a(A)	2.95 ± 0.04a(A)	2.94 ± 0.05a(AB)		2.90 ± 0.03a(AB)	2.84 ± 0.05ab(AB)	2.88 ± 0.11ab(AB)	2.49 ± 0.11b(B)
KL/KW ratio	2.05 ± 0.02a	2.04 ± 0.02a	2.00 ± 0.03a		2.10 ± 0.04a	2.10 ± 0.05a	2.13 ± 0.08a	2.19 ± 0.08a
TKW(g)	42.68 ± 0.65a(A)	41.97 ± 1.41a(A)	40.53 ± 3.09a(AB)		39.99 ± 1.11a(A)	40.30 ± 1.69a(A)	38.69 ± 3.37ab(AB)	25.09 ± 3.62b(B)
	**06LY**							
	***A/1***	***A/2***	***A/3***	***A/4***	***G/1***	***G/2***	***G/3***	***G/4***
**Overall**								
KL (mm)	6.71 ± 0.06a(A)	6.60 ± 0.10ab(AB)	6.30 ± 0.07bc(B)	6.05 ± 0.13abc(AB)	6.75 ± 0.09a(A)	6.53 ± 0.08abc(AB)	6.42 ± 0.10abc(AB)	6.17 ± 0.09c(B)
KW (mm)	3.34 ± 0.03a(A)	3.24 ± 0.03acd(AB)	3.12 ± 0.03bf(BC)	3.12 ± 0.07abf(ABC)	3.20 ± 0.04bc(ABC)	3.15 ± 0.03bdf(BC)	3.10 ± 0.02bef(BC)	3.03 ± 0.03f(C)
KT(mm)	2.93 ± 0.03a(A)	2.86 ± 0.03ab(AB)	2.76 ± 0.02bc(B)	2.83 ± 0.04abc(AB)	2.93 ± 0.03a(A)	2.85 ± 0.03abc(AB)	2.85 ± 0.02abc(AB)	2.72 ± 0.03c(B)
KL/KW ratio	2.01 ± 0.02a	2.04 ± 0.03a	2.02 ± 0.02a	1.95 ± 0.07a	2.11 ± 0.03a	2.08 ± 0.03a	2.07 ± 0.03a	2.04 ± 0.03a
TKW (g)	41.85 ± 0.84a(A)	39.09 ± 1.25acde(AC)	33.38 ± 0.83b(B)	32.48 ± 2.09cb(AB)	39.70 ± 1.28 ac(AC)	35.19 ± 1.05bd(BC)	34.57 ± 0.83be(BC)	30.89 ± 0.90bf(BD)
**Landraces**								
KL (mm)	6.52 ± 0.20a	6.33 ± 0.11a	6.26 ± 0.07a	6.05 ± 0.13a	6.72 ± 0.18a	6.40 ± 0.09a	6.42 ± 0.11a	6.18 ± 0.10a
KW (mm)	3.13 ± 0.08a	3.12 ± 0.04a	3.10 ± 0.03a	3.12 ± 0.07a	3.13 ± 0.07a	3.10 ± 0.03a	3.10 ± 0.03a	3.04 ± 0.03a
KT (mm)	2.74 ± 0.07a	2.73 ± 0.03a	2.74 ± 0.03a	2.83 ± 0.04a	2.88 ± 0.05a	2.81 ± 0.04a	2.83 ± 0.03a	2.73 ± 0.03a
KL/KW ratio	2.09 ± 0.07a	2.03 ± 0.04a	2.03 ± 0.03a	1.95 ± 0.07a	2.15 ± 0.06a	2.07 ± 0.03a	2.08 ± 0.04a	2.03 ± 0.03a
TKW (g)	34.22 ± 2.28ab	33.24 ± 1.21ab	32.69 ± 0.83ab	32.48 ± 2.09ab	37.55 ± 2.51a	32.19 ± 1.00ab	34.15 ± 0.83ab	31.27 ± 0.89b
**Modern cultivars**								
KL (mm)	6.75 ± 0.06a	6.74 ± 0.13a	6.53 ± 0.16a		6.77 ± 0.09a	6.79 ± 0.15a	6.41 ± 0.29a	6.13 ± 0.03a
KW (mm)	3.38 ± 0.02a	3.31 ± 0.04ab	3.28 ± 0.05ab		3.24 ± 0.04ab	3.25 ± 0.05ab	3.11 ± 0.03ab	2.95 ± 0.15b
KT (mm)	2.97 ± 0.02a	2.93 ± 0.03ab	2.89 ± 0.07ab		2.95 ± 0.03ab	2.95 ± 0.04ab	2.94 ± 0.06ab	2.63 ± 0.13b
KL/KW ratio	2.00 ± 0.02a	2.04 ± 0.04a	1.99 ± 0.05a		2.09 ± 0.04a	2.09 ± 0.04a	2.06 ± 0.08a	2.09 ± 0.12a
TKW (g)	43.34 ± 0.70a(A)	42.14 ± 1.47a(AB)	38.47 ± 2.49ab(AB)		40.93 ± 1.39a(AB)	41.01 ± 1.65a(AB)	37.36 ± 3.02ab(AB)	27.27 ± 4.39b(B)

02LY, Luoyang (2002); 06LY, Luoyang (2006).

Different capital and small letters indicate significant between haplotypes at *P* <0.01 and *P* <0.05.

*A/1, *Hap-6A-A/Hap-6B-1*; landraces, *N* = 8; Modern cultivars, *N* = 41.

*A/2, *Hap-6A-A/Hap-6B-2*; landraces, *N* = 13; Modern cultivars, *N* = 25.

*A/3, *Hap-6A-A/Hap-6B-3*; landraces, *N* = 37; Modern cultivars, *N* = 5.

*A/4, *Hap-6A-A/Hap-6B-4*; landraces, *N* = 5; Modern cultivars, *N* = 0.

*G/1, *Hap-6A-G/Hap-6B-1*; landraces, *N* = 12; Modern cultivars, *N* = 21.

*G/2, *Hap-6A-G/Hap-6B-2*; landraces, *N* = 31; Modern cultivars, *N* = 16.

*G/3, *Hap-6A-G/Hap-6B-3*; landraces, *N* = 26; Modern cultivar, *N* = 4.

*G/4, *Hap-6A-G/Hap-6B-4*; landraces, N = 19; Modern cultivars, N = 2.

### *TaGW2-6B* has a stronger effect than *TaGW2-6A* on TKW

Based on the haplotype polymorphisms of *TaGW2-6A* and *TaGW2-6B*, the phenotypic explanation rates (*R*^*2*^) for grain traits was calculated in the same set of 265 accessions (Table 3). In landraces, *R*^*2*^ for grain traits in *TaGW2-6B* was higher than that in *TaGW2-6A*, and the value of the combination of *TaGW2-6A*/*TaGW2-6B* was higher than that of either *TaGW2-6A* or *TaGW2-6B* alone in both growing seasons (Table 3). As for modern cultivars, the *R*^*2*^ of the combination of these two genes was still the highest, *TaGW2-6B* followed and *TaGW2-6A* was the lowest. Although they had the similar *R*^*2*^ trends in these two subpopulations, *R*^*2*^ of these haplotypes in the modern cultivars was significantly higher than in landraces, especially for KW and TKW. This further indicated that these grain trait-related genes had undergone strong positive selection in modern breeding, and that *TaGW2* controlled grain weight in terms of regulating grain width during development. In addition, the *R*^*2*^ values of the *TaGW2-6A*/*TaGW2-6B* combination were higher than those of *TaGW2-6A* plus *TaGW2-6B* for all grain traits in two environments in the landraces. However, in modern cultivars, the phenotypic effect of the combination of these two haplotypes was less than that of their simple sum.

**Table 3:** **Phenotypic explanation rates (*R***^***2***^**) of *TaGW2-6A, TaGW2-6B *** **and their combinations for grain traits in landrace and modern cultivar subpopulations in two environments**

**Item**	**02LY**			**06LY**		
	***TaGW2-6A***	***TaGW2-6B***	***TaGW2-6A/TaGW2-6B***	***TaGW2-6A***	***TaGW2-6B***	***TaGW2-6A/TaGW2-6B***
Landraces						
TKW (g)	0.44	7.17	10.40	0.09	6.19	8.52
KL (mm)	0.13	5.77	6.41	1.12	6.81	8.57
KW (mm)	3.58	3.76	9.76	0.27	1.58	2.33
KT (mm)	0.01	2.71	9.57	3.47	1.62	9.52
Modern cultivars						
TKW (g)	5.79	13.54	16.82	3.94	12.88	14.99
KL (mm)	0.26	3.23	3.49	0.03	4.84	5.04
KW (mm)	9.73	10.51	17.36	10.55	9.73	18.38
KT (mm)	8.31	12.08	17.79	0.36	8.55	8.97

02LY, Luoyang (2002); 06LY, Luoyang (2006).

## Discussion

### Natural diversity in cereal yield genes usually occurs in promoter and intron regions that influence gene expression levels

The isolation of genes controlling grain weight in wheat and development of functional markers are desirable for marker-assisted-selection (MAS) breeding. On the basis of genetic information, several successful examples of MAS combined with phenotypic measurement have been accomplished, and these have mainly focused on improvement of discontinuous traits such as resistance to pests/disease, stress tolerance, and grain quality [28-39]. Liu *et al.*[40] recently reviewed progress in functional marker development in wheat, including the 97 markers associated with processing quality, agronomic traits and disease resistance. In this review, three markers for wheat grain weight genes were also mentioned; they were *TaSus2-2B*, *TaGW2-6A* and *TaCwi-A1*[19,41,42], respectively. Among them, the CAPS marker of *TaGW2-6A* distinguished *Hap-6A-A* and *Hap-6A-G* with higher accuracy and repeatability, making it an effective marker for selection of kernel weight [43].

In some cereal yield genes, natural diversity usually occurred in promoter or intron regions, which influenced gene expression levels. For example, the differences in expression levels of *GS5* in rice are attributable to polymorphisms in the promoter region, leading to grain width variation [44]. Another good example is that one polymorphism in the promoter region of *ZmGS3* was found to affect HKW in two environments [45]. It is also found that one SNP in the promoter region of *ZmGW2-CHR4* was significantly associated with KW and HKW, and the expression level of this gene was negatively correlated with KW [18]. Further analysis of polymorphism in the *TaGW2-6B* and *TaGW2-6D* diversity in this study showed that most of the diversity existed in the promoter region of *TaGW2-6B*, with no diversity in *TaGW2-6D* (Figure 1). This is consistant with findings for yield gene diversity in rice [5] and maize [18,45]. In addition, the diversity of wheat yield gene *TaDep1* and *TaSUS1-7B* were also found in intron regions, which influenced gene expression levels [46,47].

Association analysis of grain traits suggested that *Hap-6B-1 and Hap-6B-2* were favored haplotypes (Table 1), and the *TaGW2*-6B-CAPS markers could distinguish the favored haplotypes from other haplotypes, indicating that they could be used as diagnostic markers in MAS for increased grain weight.

The average TKW of *Hap-6A-A* was about 3.1 g higher than that of *Hap-6A-G* in Chinese modern cultivars [19]. Moreover, genotypes with the *Hap-6A-A* allele had earlier heading and maturity dates of about 3.5 and 2.5 days than *Hap-6A-G* genotypes. In this study, the favored haplotype *Hap-6B-1* also affected wheat maturity, with earlier heading and maturity dates compared to other haplotypes (Additional file 3: Figure S2). This may be caused by: (1) a hitchhiking effect of a developmentally-related gene during selection of *Hap-6B-1* in domestication and breeding; genes affecting maturity have been mapped near the chromosome group 6 centromere regions [48], or (2) a pleiotropic effect of *TaGW2*. The additive effect of *TaGW2-6A* combined with *TaGW2-6B* showed that *Hap-6A-A*/*Hap-6B-1* was a superior combination conferring high TKW in modern cultivars (Table 2, Figure 8), although is effect in landraces was not. The combination effect of the two favored haplotypes was significantly higher than that of any single haplotype (Figure 8), indicating an obvious additive effect.

### Strong selection of *TaGW2-6A* and *TaGW2-6B* haplotypes occurred in global wheat breeding

In most domesticated crops, genetic diversity differs under conditions of continuous natural and artificial selection. In this process, about 2 to 4% of maize genes (a minimum of 1,200) throughout the genome were targeted for selection during domestication and improvement [49], and many genes, such as the *Waxy* gene in rice [50], height-reduction genes *Rht-B1* and *Rht-D1* in wheat [51], and the tomato fruit size gene *fw2.2*[52] had retained a selection trace. Grain weight is a quantitative trait controlled by multiple genes and is also positively selected during domestication and breeding. In modern wheat breeding, genes controlling yield traits have undergone strong artificial selection and the frequency distributions of their variations are extremely uneven, and the alleles associated with ecological adaptation and favored agronomic traits are present at high frequency [53-55].

Our previous study investigated the distribution of *TaGW2-6A* haplotypes in Chinese and European wheat cultivars [19]. In this study, the distribution of *TaGW2-6A* and *TaGW2-6B* haplotypes are systematically described for six major regional wheat production regions worldwide, viz. China, US, Canada, Russia, Australia, Europe and Mexico (Figure 6). Haplotype *Hap-6A-A* at *TaGW2-6A* was favored in cultivars released in China, Australia and Russia, whereas *Hap-6A-G* was frequent in other areas. In addition, *Hap-6A-A* was mainly distributed in spring and weak-winter wheat cultivars with early maturity, which was favorable to increasing the multi-cropping index, whereas in winter and strong-winter wheat cultivars *Hap-6A-G* was more frequent. This geographic difference in *TaGW2-6A* haplotypes may be related to the breeding and planting environment. In contrast, the distribution of favored *TaGW2-6B* haplotypes tended to be more consistent across global wheat cultivars, and favored haplotypes *Hap-6B-1* and *Hap-6B-2* have undergone strong positive selection and accumulation in breeding programs.

### *TaGW2* negatively regulates grain weight, genotypes with lower expression were positively selected in breeding

In recent years scientists have been searching for key genes controlling kernel weight in cereals because it is a major component of yield. In rice, *GS3*, *GW2* and *qSW5* negatively regulate grain size [7,8,56]. In contrast, *GW8* and *GS5* were isolated as positive regulators of grain size, and their higher expression levels could be involved in promoting cell division and ultimately increasing grain yield [10,44]. A comparative genomics study found that the expression level of the *ZmGW2-CHR4* gene was negatively correlated with grain weight in maize [18], and shared the same regulation pattern as *GW2* in rice. Gene *TaGW2* regulated grain size in wheat through variation in expression level [19]. SNPs in the *TaGW2-6A* promoter region may be related to expression of different alleles, such as *Hap-6A-A* and *Hap-6A-G*. The regulation pattern of *TaGW2-6A* was consistent with *OsGW2*, both of which negatively regulated grain width and weight [8]. Yang *et al.*[21] identified a 1 bp insertion at the 977th base pair of *TaGW2-6A* in cultivar Lankaodali and a derived SNP marker was used to genotype an F_2_ population derived from a cross of Lankaodali (TT) and Chinese Spring (tt). Compared with the tt genotype, the average increase in TT genotypes was 0.18 mm for KW and 3.94 g for TKW, indicating that *TaGW2-6A* regulated grain width and grain weight. However, Bednarek *et al.*[22] showed that RNAi resulted in down-regulation of *TaGW2* expression in wheat, inducing significant decreases in grain parameters (viz. final grain fresh, dry and irrigation masses, and grain volume, width, and thickness). They concluded that *TaGW2* positively regulated grain size in wheat. For relative expression analysis RT-PCR was performed at different seed development periods after flowering in the present study (Additional file 6: Figure S5), in order to objectively evaluate the relationship between gene expression and grain size/weight. Our results showed that the relative expression levels of the *TaGW2-A/B/D* orthologs in developing seeds were all negatively correlated with grain width/weight.

The *cis*-elements in promoter regions of *TaGW2-6A* and *TaGW2-6B* were predicted by Plantcare. More *cis*-elements were found on *Hap-6A-A* than on *Hap-6A-G*. Due to the SNP at -2070 bp, there is an endosperm tissue-specific expression element GCN4_motif in *Hap-6A-A*, but in corresponding region of *Hap-6A-G,* it is an ABA response element, ABRE (Figure 2). For *TaGW2-6B* locus*,* at -929 bp, a G-box was detected in *Hap-6B-3* and *Hap-6B-4*, an auxin responsive element (AuxRR-core) was found in *Hap-6B-1* at -1709 bp, an ABRE was detected in *Hap-6B-2*, *Hap-6B-3* and Hap-6B-4 (Figure 2). The influence of these *cis*-elements on *TaGW2* expression need to be further elucidated in the future.

Moreover, the average relative expression level of *TaGW2-6A* was higher than that of *TaGW2-6B* and *TaGW2-6D*, and the phenotypic explanation rate (*R*^*2*^) for grain traits explained by *TaGW2-6B* was higher than that of *TaGW2-6A* in both landraces and modern cultivars (Table 3). Therefore, the lower the relative expression level of a *TaGW2* gene, the higher its *R*^*2*^ value, indicating that grain size is negatively regulated by *TaGW2* genes. Thus among the three *TaGW2* homoeologous genes, the average expression of *TaGW2-6A* during the grain development was the highest, but its *R*^*2*^ was the lowest. *TaGW2-6D* gene was the most conservative with the lowest mean expression among the three homoeologous genes, and its *R*^*2*^ was estimated to be the highest. There are three possible reasons for lack of diversity at *TaGW2-6D* locus. Firstly, during evolution from tetraploid to hexaploid wheat, the increase in KW was larger than that in KL. Secondly, the D genome has much lower diversity than the A- or B- genomes in common wheat [57,58]. Thirdly, *TaGW2-6D* has the strongest effect on TKW, and it underwent strong positive selection and fixation in early domestication of hexaploid wheat (Table 3) [59].

## Conclusions

Haplotypes, expression, genetic effects and geographic distribution of wheat yield gene *TaGW2* were analyzed. Major variations occurred at their promoter regions in the three homoeologous genes. Expression levels of *TaGW2*s were negatively correlated with TKW, which further supported earlier conclusion that the *GW2* negatively regulates grain size in cereals. Haplotype interaction analysis exhibited the additive effects between favored haplotypes at *TaGW2-6A* and *TaGW2-6B*. We also found that haplotypes at *TaGW2-6A* and *TaGW2-6B* underwent strong selection in one century of global wheat breeding. Therefore, there are major genes even though yield is a complex quantitative trait. It illustrated that association based on haplotypes is more effective than single marker in dissection of complex traits. This study provided important genes and functional markers for MAS in wheat yield improvement.

## Methods

### Plant materials

Four hundred and seventy one Chinese wheat accessions including 151 landraces and 320 modern cultivars were used for functional validation of the *TaGW2-6B* markers (Additional file 9: Table S1), among which 265 accessions (151 landraces and 114 modern cultivars) were mainly from the Chinese wheat mini-core collection (MCC) representing more than 70% of the genetic diversity of the total Chinese germplasm collection [24]. Association analysis between *TaGW2-6A* markers and grain traits earlier confirmed that these materials provided good representation and effectiveness when used as a population for association analysis [19,60,61]. In addition, 1,032 introduced wheat cultivars comprising 374 European, 471 American, 51 Australian, 53 CIMMYT and 83 Russian accessions were used for haplotype distribution analysis of *TaGW2-6A* and *TaGW2-6B* in global wheat cultivars (Additional file 10: Table S2).

Chinese Spring was used to clone the promoter sequences of *TaGW2-6B* and *TaGW2-6D*. A set of Chinese Spring (CS) nullisomic-tetrasomic lines was used for chromosome location of *TaGW2*. Thirty-four accessions with large variations in grain weight, including 22 modern cultivars and 12 landraces (Additional file 1: Table S3), were used for sequencing to detect single-nucleotide polymorphisms (SNPs) and haplotypes in the promoter regions of *TaGW2-6B* and *TaGW2-6D*.

A recombinant inbred line (RIL) population derived from Nanda 2419 and Wangshuibai was used for fine mapping of *TaGW2-6B*.

### Measurement of grain weight related traits

During the 2001-2002 and 2005-2006 wheat-growing seasons, cultivars used in this study were planted at the CAAS-Luoyang Experiment Station in Henan province (111.6°E, 33.8°N). Each cultivar was planted in 2 m double rows spaced 25 cm apart, with 40 seeds planted in each row. The field management followed local practices.

After harvest, 20 grains were randomly selected from each genotype and lined up length-wise along a ruler to measure average kernel length (KL), and then arranged breadth-wise to measure kernel width (KW). The middle parts of ten grains were measured with vernier calipers to estimate average kernel thickness (KT). Two independent samples of 500 grains were weighted and the means were converted to one thousand-kernel weight (TKW).

### Cloning the promoters of *TaGW2-6B* and *TaGW2-6D*

Genomic DNA was extracted from young leaves of 10-day-old seedlings using a modified CTAB method [62]. Primers were designed by the software Primer Premier Version 5.0 (Premier Biosoft International, Palo Alto, CA), and all primers were synthesized by Shanghai Sangon Biological Technology Co., Ltd (http://www.sangon.com/).

Primers pF147 and pR1481 were designed to amplify the 1.5 kb promoter regions of *TaGW2-6B* and *TaGW2-6D* according to the *TaGW2-6A* promoter sequence. PCR were performed in total volumes of 15 μl, including 3 pmol of each primer, 120 μM of each dNTP, 80 ng genomic DNA, 0.75 unit La-Taq and 7.5 μl of 2 × GC Buffer (TaKaRa Biotechnology (Dalian) Co. Ltd, Product Code: DRR20AG). PCR were performed as follows: 95°C for 4 min; followed by 35 cycles of 95°C for 30 s, annealing (60-64°C) for 30 s, and extension at 72°C (30 s to 3 min), and 72°C for 30 s, with a final extension of 72°C for 10 min. The annealing temperatures and extension times depended on the primer sets and the lengths of the expected PCR products. The PCR products were separated by electrophoresis in agarose gels, and the target bands were extracted and cloned into the pEASY-T1 simple vector and transformed to DH5α competent *E. coli* cells by the heat shock method (Beijing Trans Gen Biotech Co., Ltd, Product Code: CT111). Positive clones were selected for sequencing by ABI 3730XI DNA Analyzer. To guarantee sequence accuracy, the PCR and DNA sequencing were repeated at least three times.

A Genome Walking Kit (TaKaRa Biotechnology (Dalian) Co. Ltd, Product Code No. 6108) was used to obtain the 5′ flanking promoter sequence of *TaGW2-6B* and *TaGW2-6D*. The kit was based primarily on the known genomic DNA sequence, utilizing the annealing temperature difference between degenerate primers and specific primers for thermal asymmetric PCR, and flanking sequences were obtained by three nested PCR. Three specific primers TaGW2-SP1, TaGW2-SP2 and TaGW2-SP3 (Additional file 11: Table S4) were designed according to the promoter sequence to amplify the sequences in the unknown regions. Here, TaGW2-SP2 was designed on the inside of TaGW2-SP1, and TaGW2-SP3 was located on the inside of TaGW2-SP2. Each distance between the two primers was 60-100 bp. Three PCR were performed in total volumes of 25 μl, including 80 ng genomic DNA, 4 μl of 2.5 mM dNTP mixture, 2.5 μl of 10 × LA PCR Buffer II (Mg^2+^ plus), 1.25 U of TaKaRa LA Taq, 0.5 μl of AP Primer (100 pmol/μl), 0.5 μl of SP Primer (10 pmol/μl). The first PCR was as follows: Taking Chinese Spring DNA as the template, AP Primer as the upstream primer, and TaGW2-SP1 as downstream primer for the first PCR. PCR were performed as follows: 94°C for 1 min, 98°C for 1 min; followed by 5 cycles of 94°C for 30 s, 62°C for 1 min, 72°C for 2 min; 94°C for 30 s, 25°C for 3 min, 72°C for 2 min; followed by 15 cycles of 94°C for 30 s, 62°C for 1 min, 72°C for 2 min, 94°C for 30 s, 62°C for 1 min, 72°C for 2 min, 94°C for 30 s, 44°C for 1 min, 72°C for 2 min; and 72°C for 10 min. The second PCR was performed as follows: the first PCR solution was diluted 100 times, taking 1 μl as template for the second PCR, AP primer as the upstream, and TaGW2-SP2 as the downstream primer. PCR were performed as follows: 15 cycles of 94°C for 30 s, 63°C for 1 min, 72°C for 2 min, 94°C for 30 s, 63°C for 1 min, 72°C for 2 min, 94°C for 30 s, 44°C for 1 min, 72°C for 2 min; and 72°C for 10 min. For the third PCR, the second PCR solution was diluted 100 times, then taking 1 μl as the template, AP primer as the upstream primer, and TaGW2-SP3 as the downstream primer. PCR were performed as for the second PCR. The three PCR products were separated by electrophoresis in 1% agarose gels; the target bands were extracted and PCR products were selected for sequencing using the primer TaGW2-SP3. A sequence of about 700 bp was obtained by genome walking, taking this sequence as a reference to continue amplifying upstream sequences. Through this method of genome walking, ~2.9 Kb of promoter sequences of *TaGW2-6B* and *TaGW2-6D* were obtained.

### SNP identification and functional marker development

Thirty-four cultivars were initially chosen for detecting sequence variation in the promoter regions of *TaGW2-6B* and *TaGW2-6D*. SNPs were identified using DNA Star software (http://www.dnastar.com/). The core elements of the promoters were identified using the TSSP program (http://www.softberry.com). The cis-acting regulatory elements of the promoter were predicted by Plantcare (http://bioinformatics.psb.ugent.be/webtools/plantcare/html/).

Four haplotypes formed by 11 SNPs were found in *TaGW2-6B* and three markers were developed to distinguish them. Genome-specific primers were designed for these three markers, respectively (Additional file 11: Table S4). Basically, the three systems of PCR and digestion were similar. PCR were performed in total volumes of 15 μl, including 80 ng genomic DNA, 1.5 μl of 10 × PCR Buffer, 1.5 μl of MgCl_2_ (25 mM), 10 μM of each primer, 0.16 μl of dNTP (25 μM), 0.75 U of Taq (Fermentas). PCR were performed as follows: 95°C for 4 min; followed by 35 cycles of 95°C for 30 s, annealing (60-64°C) for 30 s, and extension at 72°C (30 - 90 s), and 72°C for 30 s, with a final extension of 72°C for 10 min. The annealing temperatures and extension times depended on the primer sets and the lengths of expected PCR products (Additional file 11: Table S4). Digestions were performed in total volumes of 10 μl, including 5 μl of PCR products, 1 μl of 10 × Buffer, 0.1 μl of BSA, 0.25 μl of restriction enzyme, and 3.65 μl of ddH_2_O.

The *TaGW2*-6B-CAPS marker amplified a 1.4 Kb genome-specific fragment, which was digested with BstNI (NEB) according to the manufacturer’s instructions, and the digested segments were separated on 1.5% agarose gels with EB. This marker effectively distinguished *Hap-6B-1* and three other haplotypes. *Hap-6B-1* (no restriction sites) was represented by a single 1.4 kb band, whereas the other three were digested into 1.0 Kb and 400 bp bands.

The *TaGW2*-6B-ACAS marker needs two PCR amplifications, the *TaGW2*-6B-ACAS-1 primer amplified a 464 bp fragment and the *TaGW2*-6B-ACAS-2 primer amplified a 626 bp fragment. The two fragments represent *Hap-6B-2* and other two haplotypes, respectively; they were easily distinguished in 1.5% agarose gels.

The *TaGW2*-6B-dCAPS marker amplified a 263 bp genome-specific fragment; the products were then digested with Hpy166II (NEB) at 65°C for 20 min, and the digested segments were separated on 8% non-denaturing polyacrylamide gels. The marker distinguished *Hap-6B-3* (263 bp) and *Hap-6B-4* (240 bp).

### RNA extraction, reverse transcription and real-time quantitative PCR

For each cultivar, spikes from all tillers of each individual were tagged at anthesis when the first spikelets on the head flowered, and the date was recorded. Grains were harvested at 5, 10, 15, 20, 25 and 30 days post-flowering (dpf) as well as mature seeds. Genome-specific primers were designed according to cDNA sequence differences of the *GW2* homoeologues from chromosomes 6A, 6B and 6D to evaluate the correlation of gene expression levels of *TaGW2-6A/6B/6D* and grain weight. To investigate the temporal expression profiles of the *TaGW2s* during grain development, RT-PCR was conducted using developing seeds at 5, 10, 15, 20, 25, and 30 days post-flowering (dpf) and mature seeds from 22 wheat cultivars including 12 small grained and 10 large grained genotypes. mRNA was extracted using TIANGENRNA Plant Plus Reagent. cDNA was synthesized with the SuperScript II System (Invitrogen) according to the manufacturer’s instructions. DNA was removed by digestion with DNaseI (Fermentas) before reverse transcription. The expression analysis of *TaGW2* was performed with SYBR Premix Ex-Taq [TaKaRa Biotechnology (Dalian) Co. Ltd, Product Code: DRR041A]. RT-PCR were performed in total volumes of 2 0 μl, including 1 μL cDNA, 10 μl of 2× SYBR Premix Ex-Taq, 0.4 μl of each primer (10 μM) and 0.4 μl of ROX Reference Dye (50×). The primer sets of *TaGW2-6A*, *TaGW2-6B*, *TaGW2-6D* and Actin (Additional file 11: Table S4) were used for amplification of *TaGW2* and actin genes, respectively. Three replications were performed to obtain average values and standard deviations of expression level for each gene. Cts were exported and averaged from technical triplicates. Relative expression was determined using the *ΔCt* method corrected for primer efficiencies. Actin gene was used as endogenous control, which was not changed too much in different tissues and developmental stages of wheat under our experiment. This housekeeping gene was assayed on our experimental samples and data were normalized to the expression of actin. To compare *TaGW2* homoeologous expression, the relative expression values of *TaGW2s* were calculated using the *2-ΔΔCt* method [63], with the 5 dpf stage of Chinese Spring as a reference sample for *ΔΔCt*.

### Statistical analyses

Statistical analyses were based on phenotypic data of average grain size and grain weight in two environments. Variance analyses were performed on the SPSS System for Windows version 12.0 to determine phenotypic differences between the four haplotypes individually and in haplotype combinations, based on analysis of variance (One-Way ANOVA) according to Tukey test at the significance level of 5% (*P* ≤ 0.05). *TaGW2-6B* was mapped using data from recombinant inbred lines derived from the cross between Nanda 2419 and Wangshuibai using MAPMAKER/EXP 3.0 [64]. Phenotypic explanation rate (*R*^*2*^) of *TaGW2-6A* and *TaGW2-6B* was the ratio between the sum of squares between groups and total squares for the various haplotypes.

## Abbreviations

L: Landraces; M: Modern cultivars; TKW: One-thousand kernel weight (g); KL: Kernel length (mm); KW: Kernel width (mm); KT: Kernel thickness (mm); MCC: Chinese mini core collection.

## Competing interests

The authors declare that they have no competing interests.

## Authors’ contributions

QL carried out the molecular genetic studies, participated in the sequence alignment and drafted the manuscript. HCY participated in the design of the study, performed data analysis and participated in tables and figures. ZXY, MZQ supervised the study and revised the manuscript critically. HJ, WYQ, LT, WLF collected data and measured grain weight related traits*.* All authors read and approved the final manuscript.

## Supplementary Material

Additional file 1: Table S3 Wheat accessions used for obtaining of promoter sequences. TKW, One-thousand kernel weight (g), KL, kernel length (mm), KW, kernel width (mm), KT, kernel thickness (mm). *: accessions used for real-time PCR analysis of *TaGW2s*. Click here for file

Additional file 2: Figure S1 Genetic mapping of *TaGW2-6B* in a Nanda2419 × Wangshuibai recombinant inbred line (RIL) population. *TaGW2-6B* gene (red marker) was mapped between *Xmag359* and *Xwmc341* on chromosome 6B. The left side of the map was map distance/cM, the right side was SSR markers. Click here for file

Additional file 3: Figure S2 Differences on heading and maturity dates among *TaGW2-6B* haplotypes in landraces and modern cultivars based on two years of field data. A, Heading dates differences among *TaGW2-6B* haplotypes in 2002 and 2006. B, Maturity dates differences among *TaGW2-6B* haplotypes. The bars represent the standard deviation. Click here for file

Additional file 4: Figure S3 Haplotype frequencies (%) of *TaGW2-6A* and *TaGW2-6B* in wheat cultivars released since the 1950s in China, Europe and America. A, the frequency of *Hap-6A-A*; B, the frequency of *Hap-6A-G*; C, the frequency of *Hap-6B-1*; D, the frequency of *Hap-6B-4*. Click here for file

Additional file 5: Figure S4 Mean relative expressions of *TaGW2-6A, -6B, -6D* in two subgroups. Red lines represent the subgroup with higher TKWs (10 accessions, mean TKW 47.2 g) and blue lines represent the subgroup with lower TKWs (12 accessions, mean 28.5 g). A, mean relative expressions of *TaGW2-6A*; B, mean relative expressions of *TaGW2-6B*; C, mean relative expressions of *TaGW2-6D. TaGW2* genes negatively regulate grain size. Normalized values of *TaGW2* (relative expression) are given as Mean ± SD. Click here for file

Additional file 6: Figure S5 Mean relative expressions of haplotypes in *TaGW2-6A* at different grain development stages based on 22 wheat accessions. Normalized values of *TaGW2* (relative expression) are given as Mean ± SD. Click here for file

Additional file 7: Figure S6 TKW and KW for eight haplotype combinations of *TaGW2-6A* and *TaGW2-6B* in landraces and modern cultivars grown in two environments. A, TKW for eight haplotype combinations in landraces; B, KW for eight haplotype combinations in landraces; C, TKW for eight haplotype combinations in modern cultivars; D, KW for eight haplotype combinations in modern cultivars; *A*/*1*, *Hap-6A-A*/*Hap-6B-1*; *A*/*2*, *Hap-6A-A*/*Hap-6B-2*; *A*/*3*, *Hap-6A-A*/*Hap-6B-3*; *A*/*4*, *Hap-6A-A*/*Hap-6B-4*; *G*/*1*, *Hap-6A-G*/*Hap-6B-1*; *G*/*2*, *Hap-6A-G*/*Hap-6B-2*; *G*/*3*, *Hap-6A-G*/*Hap-6B-3*; *G*/*4*, *Hap-6A-G*/*Hap-6B-4*. Click here for file

Additional file 8: Figure S7 Frequencies of *TaGW2-6A* and *TaGW2-6B* haplotype combinations in Chinese wheat landraces and modern cultivars. Click here for file

Additional file 9: Table S1 Accessions used for association analysis and haplotype distribution studies in Chinese wheat collections. L, landraces; M, modern cultivars; TKW, One-thousand kernel weight (g); KL, kernel length (mm); KW, kernel width (mm); KT, kernel thickness (mm); *, Chinese mini core collection; I, Northern winter wheat region; II, Yellow and Huai River valley winter wheat region; III, low and middle Yangtze River valley winter wheat region; IV, southwestern winter wheat region; V, southern winter wheat region; VI, northeastern spring wheat region; VII, northern spring wheat region; VIII, northwestern spring wheat region; IX, Qinghai-Tibet spring-winter wheat region; X, Xinjiang winter-spring wheat region. Click here for file

Additional file 10: Table S2 Accessions used for geographic distribution analysis of haplotypes in global wheat cultivars. Click here for file

Additional file 11: Table S4 Primer sequences used in this study. Click here for file
