# Supplementary material for: Homologous haplotypes, expression, genetic effects and geographic distribution of the wheat yield gene TaGW2
Source: BMC Plant Biol. 2014 Apr 25;14:107. doi: 10.1186/1471-2229-14-107 (PMC4021350; doi:10.1186/1471-2229-14-107)
Supplement: Additional file 11: Table S4 — Primer sequences used in this study. [file 1471-2229-14-107-S11.doc]

**Table S4** Primer sequences used in this study.

| Primer set | Primer sequence | Amplified target | Tm (°C) |
| --- | --- | --- | --- |
| pF147 | CGTTACCTCTGGTTTGGGTGTCGTG |  | 60 |
| pR1781 | GCGGCACTCTACGGCAGAACAAAT |  |  |
| TaGW2-SP1 | GGGTCCAGGGGCTTTACAAATGAC |  | 62 |
| TaGW2-SP2 | CCATTGCTGGAGGAACTAGACAAAC |  | 63 |
| TaGW2-SP3 | GCATCAAGGGGATGAGCAACATAGG |  | 63 |
| TaGW2-6B-CAPS | Forward: GACTCCTCCTCGTCACCCATAAAGT | CAPS marker | 64 |
|  | Reverse: ATAGCACCAGCCCTTTCTCTTC |  |  |
| TaGW2-6B-dCAPS | Forward: TGATGAGATCCCGTGCAGTAGTTC | dCAPS marker | 64 |
|  | Reverse: CCCTTTCATCTTTCATCCAAGCA |  |  |
| TaGW2-6B-ACAS-1 | Forward: GTAGGGATCACGTATTTGCTTGGAT | ACAS PCR | 61 |
|  | Reverse: CCGCCTCACTGTAGATACATATGA |  |  |
| TaGW2-6B-ACAS-2 | Forward: CTAGCTCACGCCAGGAGAGAGAA | ACAS PCR | 62 |
|  | Reverse: CCGCCTCACTGTAGATACATAAGG |  |  |
| TaGW2-6A-RT | Forward: CTGCGGAAAGTTCACCAGATAG | TaGW2-6A Real-time PCR | 63 |
|  | Reverse: TGTCAGCAAAAGGCAACGGTA |  |  |
| TaGW2-6B-RT | Forward: GATAGCTGGAGCGGGATAGCAT | TaGW2-6B Real-time PCR | 63 |
|  | Reverse: TCAGTAACAGGCAACGGTGGAG |  |  |
| TaGW2-6D-RT | Forward: GTATAGGAAATCCTGCTTGTGGG | TaGW2-6B Real-time PCR | 63 |
|  | Reverse: TGTAAGAGAAATCCATGCTTGC |  |  |
| Actin | Forward: GGCACTGGAATGGTCAAGGCTGGTT | Actin | 63 |
|  | Reverse: TGCTTGAGCTTCATCGCCCACATAG |  |  |
| TaGW2-1 | Forward: AGTATCGTGGTGTAAAGACAAAGG | Genomic DNA  contamination | 57 |
|  | Reverse: CGAGTATGCCTAGAATGGAAAGA |  |
